# HER-2 expression in biopsy and surgical specimen on prognosis of osteosarcoma: A systematic review and meta-analysis of 16 studies

**DOI:** 10.1097/MD.0000000000003661

**Published:** 2016-06-10

**Authors:** Qingyu Zhang, Fanxiao Liu, Bomin Wang, Zhenfeng Li, Dongsheng Zhou, Qiang Yang, Jinlei Dong, Jianmin Li

**Affiliations:** a Department of Orthopedics, Qilu Hospital of Shandong University, Jinan, Shandong, China; b Department of Orthopedics, Shandong Provincial Hospital Affiliated to Shandong University, Jinan, Shandong, China; c Department of Orthopedics, Qilu Hospital, Shandong University, Jinan, Shandong, China.

**Keywords:** hER-2, meta-analysis, osteosarcoma, prognosis

## Abstract

Numerous original clinical studies have attempted to investigate the prognostic value of HER-2 overexpression in osteosarcoma, but the results of these studies are not consistent. This meta-analysis and systematic review was performed to further assess the correlation between HER-2 expression and prognosis in patients with osteosarcoma. A detailed search of relevant publications was conducted using 7 electronic databases: PubMed, Embase, the Cochrane library, the Wanfang database, the China National Knowledge Internet (CNKI) database, the Chinese VIP database, and the Chinese Biological Medical (CBM) Database for publications through August 1, 2015, using the following keywords (HER-2 OR ErbB-2 OR C-erbB-2 OR neu) AND (osteosarcoma OR osteogenic tumor). The bibliographies of potentially relevant articles and identified articles were then searched by hand. Eligible studies were those that enrolled participants with osteosarcoma and provided survival outcome in HER-2 positive and negative groups. The hazard ratio (HR) and 95% confidence interval (CI) for each individual study was calculated and pooled to obtain integrated estimates, using random effects modeling. Sixteen studies involving 934 participants with osteosarcoma met our inclusion criteria. HER-2 overexpression was documented in 42.2% of patients with osteosarcoma. Compared with patients without HER-2 overexpression, those overexpressing HER-2 had decreased overall survival (HR = 2.03, 95% CI: 1.36–3.03, *P* < 0.001). Statistical associations between HER-2 overexpression and unfavorable overall survival (OS) were observed for both biopsy and surgical removal specimens (HR = 2.07, 95%CI: 1.16–3.72, *P* = 0.014; and HR = 2.02, 95%CI: 1.10–3.71, *P* = 0.024). Results for disease-free survival (DFS) were similar. Overexpression of HER-2 is significantly associated with poor outcome for patients with osteosarcoma and should be assessed at diagnosis and after surgery as a prognostic factor. However, larger-scale multicenter clinical studies are needed to further support these findings.

## 1 Introduction

Osteosarcoma is the most frequent primary malignant bone tumor. With the introduction of high-dose chemotherapy and surgical progress, the 5-year overall survival (OS) for osteosarcoma has reached 65% to 70%.^[1]^ However, further improvements in osteosarcoma survival have not been made in the past 2 decades. Given this lack of progress, numerous studies were performed to investigate factors involving the progression and metastasis of osteosarcoma, such as C-reactive protein levels and Ezrin expression.^[2,3]^ Prognostic factors could not only identify patients at high risk for recurrence, prompting them to adopt more intensive therapy in early stage disease, but also form the basis of interventions using targeted therapies.

Human epidermal growth factor receptor 2 (HER-2), also called C-erbB2, MAC117 or neu, is a transmembrane tyrosine kinase receptor. Overexpression of HER-2 is found in ∼15% to 20% of patients with breast cancer and is associated with poor prognosis. On the other hand, therapies targeting HER-2, such as trastuzumab, have greatly extended the survival of patients with breast cancer that overexpresses HER-2.^[4,5]^

In vitro data have demonstrated overexpression of HER-2 in osteosarcoma cell lines. When treated with trastuzumab, SaoS-2, an established osteosarcoma cell line, manifested growth inhibition similar to that observed for HER-2-positive breast cancer cell lines.^[6]^ Nevertheless, studies investigating HER-2 expression in patient samples reported conflicting results.

The prognostic effect of HER-2 overexpression at diagnosis was first described almost 20 years ago.^[7]^ Methodological variability existed in subsequent studies, including the antibody and cutoff used to determine HER-2 positivity and the source of specimens. Some studies failed to identify overexpression of HER-2 in osteosarcoma, some demonstrated that HER-2 overexpression was significantly associated with less favorable outcome, and others suggested a correlation between overexpression and better prognosis.

On this issue, a previous meta-analysis was insufficient to support the prognostic value of HER-2 overexpression, and the most recently published meta-analysis of 8 studies failed to include several studies that could be pooled into a meta-analysis, especially those that correlated overexpression of HER-2 in osteosarcoma with a favorable prognosis. Furthermore, this study calculated risk estimates using a less robust method, leading to an uncertain conclusion.^[8–10]^ Therefore, we included newly published studies, adopted a more accurate statistical method, and conducted an updated meta-analysis, to further investigate the significance of HER-2 overexpression on the prognosis of osteosarcoma.

## 2 Method

### 2.1 Search process

A computerized search of all potentially eligible studies was performed in the PubMed, Embase, Cochrane library, Wanfang, CNKI, Chinese VIP, and CBM databases (up to August 1, 2015) using the following terms: (HER-2 OR ErbB-2 OR C-erbB-2 OR neu) AND (osteosarcoma OR osteogenic tumor) by 2 investigators (FX Liu and QY Zhang) independently and was checked repeatedly. No language limitations were imposed. The bibliographies of relevant articles were searched by hand to retrieve any potentially eligible studies.

### 2.2 Inclusion and exclusion criteria

Studies eligible for our meta-analysis had to conform to the following criteria: (1) the study included patients diagnosed with osteosarcoma, (2) the expression of HER-2 in tumor tissue was evaluated, (3) adequate data were provided to calculate the HR and its 95% CI for OS or disease-free survival (DFS), and (4) the publication included a prospective or retrospective cohort study. If studies contained overlapping data, the one with the most comprehensive data or published most recently was included.

### 2.3 Data extraction

The following information was extracted from original articles and entered to a standardized excel file: first author's surname, year of publication, original country, number and characteristics of participants, technique used to quantify HER-2, cutoff to determine HER-2 positivity, and inclusion period. Survival data were extracted from original articles, estimated by Tierney's method, or calculated from individual patients’ data.^[11]^ Any disagreement was resolved by discussion or help from an arbiter if necessary (ZF Li).

### 2.4 Quality analysis

The methodological quality of enrolled articles was assessed using the Newcastle–Ottawa Quality Assessment Scale (NOS), which consists of 9 items for cohort studies. Each items provided counted for 1 point in the score.^[12]^ Articles with scores ≥7 were considered to have high-quality reporting.

### 2.5 Statistical analysis

The relative frequency of death or disease recurrence in the HER-2 positive and negative groups was presented as a hazard ratio (HR) with its 95% confidence interval (CI). Risk estimates of the included studies were then pooled using random effects modeling. The between-study heterogeneity was assessed quantitatively using the *I*^2^ statistic. The heterogeneity was considered to be not statistically significant with an *I*^2^ of ≤ 50%. Subgroup analyses were performed to detect the source of heterogeneity and investigate the significance of HER-2 in different population. We further conducted sensitivity analyses to substantiate the stability of results and detect the potential source of heterogeneity. Publication bias was evaluated qualitatively by inspecting funnel plots and quantitatively through Egger's test. A 2-sided *P* value of < 0.05 in Egger's test implies a statistically significant publication bias. All statistical analyses were performed by using STATA, version 12.0 (StataCorp, College Station, TX).

Because data were extracted from published literature, informed consent or ethical approval was not required for this study. Our investigation process conformed to the Preferred Reporting Items for Systematic Reviews and Meta-Analyses (PRISMA) statement.^[13]^

## 3 Result

### 3.1 Selection process

Eventually, 16 studies with 934 participants were entered into the statistical analysis, 6^[14–19]^ in Chinese and 10^[6,7,20–27]^ in English.

**Figure 1:**
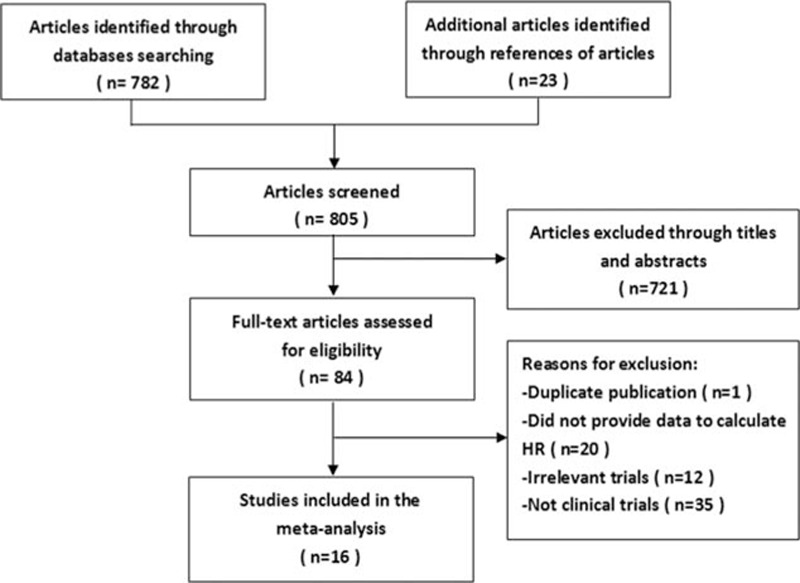
Selection process for eligible studies included in the meta-analysis.

A total of 782 potentially eligible articles were retrieved after the original search of 7 electronic databases, and 23 additional articles were retrieved from the references of relevant articles (meta-analyses, systematic reviews, letters, editorials, and guidelines). We excluded 721 apparently ineligible articles (not relevant to our investigation, reviews, and meta-analyses) by screening the titles and abstracts. After reading the full text of the remaining articles, 1^[28]^ was excluded as a duplicate publication; 47 because they were irrelevant studies or not clinical studies and 20 for lack of sufficient data to calculate HRs. The study selection process is presented as a flowchart in Fig. 1.

### 3.2 Study characteristic and quality assessment

The detailed characteristics of enrolled studies are summarized in Table 1. These studies were published from 1996 to 2014. The sample sizes of these studies ranged from 25 to149. Of 16 included studies, 10^[6,7,20–27]^ investigated tumor tissue from biopsy samples at diagnosis, whereas 6^[14–19]^ evaluated samples removed at surgery. HER-2 overexpression was documented in 42.2% of patients. Among these studies, HER-2 positivity was evaluated as 12.5% to 13.4% in 2^[20–21]^ studies (12.5%), as 30.8% to 32.1% in 2^[6,22]^ studies (12.5%), as 42.3% to 49.3% in 7^[7,16–18,23–24,27]^ studies (43.8%), as 55.3% in 1^[15]^ study (6.3%) and as 61.5% to 65% in 4^[14,19,25–26]^ studies (25%). Only 1^[20]^ used a prospective design; the others used a retrospective cohort design. The NOS scores of quality ranged from 7 to 8 with a mean of 7.625.

**Table 1:**
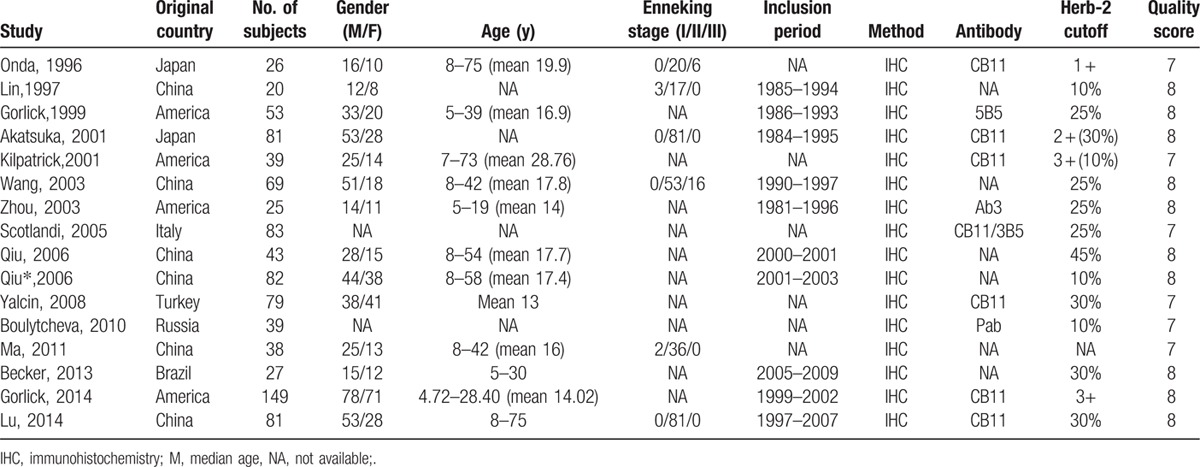
Characteristics of included studies in the meta-analysis.

### 3.3 HER-2 overexpression and survival of osteosarcoma

**Figure 2:**
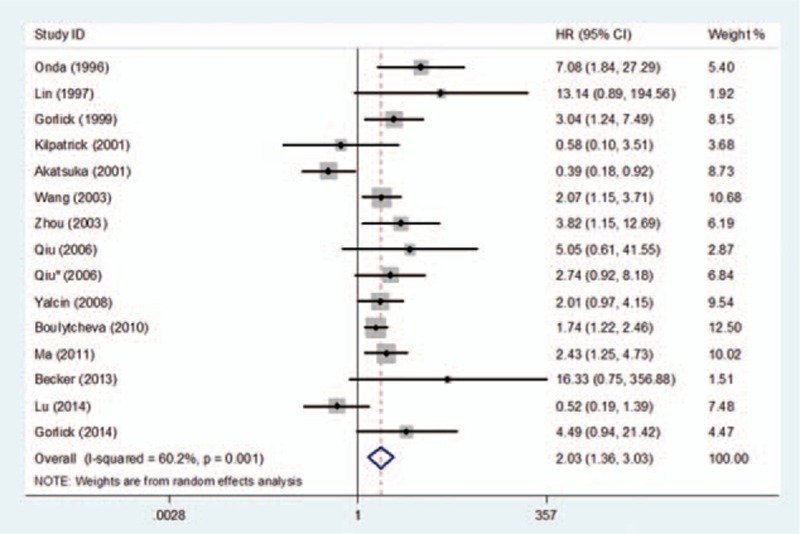
Forest plot of the association between HER-2 overexpression and overall survival in osteosarcoma.

There were 15 studies^[7,14–27]^ that reported data for overall survival (OS). The aggregated results suggested that, compared with tumors without HER-2 overexpression, those overexpressing HER-2 were significantly associated with poor OS (HR = 2.03, 95%CI: 1.36–3.03, *P* < 0.001) (Fig. 2). Statistically significant heterogeneity was found between studies (Cochran's Q *P* = 0.001, *I*^2^ = 60.2%). Background, materials, and method of all included studies were homogeneous. However, we noticed that this study (Akatsuka, 2002)^[25]^ possessed an rather higher positive rate compared with other ones. Subgroup without Akatsuka et al.'s study^[25]^ shown no statistically significant heterogeneity (Cochran's Q *P* = 0.077; *I*^2^ = 37.5%) and the result was still maintained (HR = 2.26; 95% CI: 1.62–3.15; *P* < 0.001).

**Figure 3:**
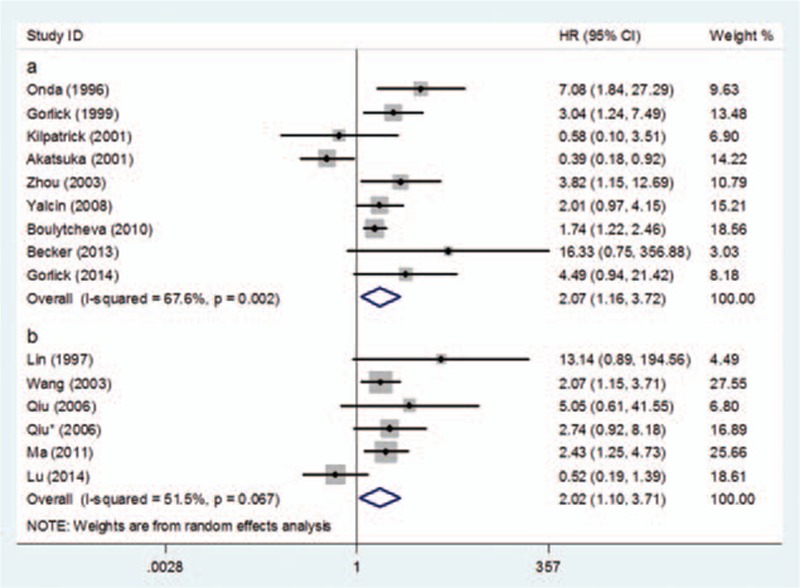
Forest plot of the subgroup analysis of OS: (A) biopsy samples and (B) surgical removal samples.

Subgroup analyses for OS were performed based on the source of samples. A total of 9 studies evaluating HER-2 expression status in biopsy specimens reported data for OS. Compared with tumors without HER-2 overexpression, those overexpressing HER-2 were significantly associated with poor OS (HR = 2.07, 95%CI: 1.16–3.72, *P* = 0.014) (Fig. 3A). Statistically significant heterogeneity was found between studies (Cochran's Q *P* = 0.002, *I*^2^ = 67.6%). Similarly, a further subgroup analysis without Akatsuka et al's study^[25]^ did not show statistically significant heterogeneity (Cochran's Q *P* = 0.167; *I*^2^ = 32.7%) and sustained the significance of the pooled estimate (HR = 2.48; 95% CI: 1.61–3.82; *P* < 0.001).

A total of 6 studies evaluating HER-2 expression status in surgical removal specimen reported data for OS. Compared with tumors without HER-2 overexpression, those overexpressing HER-2 were significantly associated with poor OS (HR = 2.02, 95% CI: 1.10–3.71, *P* = 0.024) (Fig. 3B). Statistically significant heterogeneity was also found between studies (Cochran's Q *P* = 0.067, *I*^2^ = 51.5%). Similarly, study of Lu also possessed an extremely high positive rate in this subgroup. A further subgroup analysis without Lu's study eliminated the heterogeneity (Cochran's Q *P* = 0.680; *I*^2^ = 0%) and yielded similar results (HR = 2.44; 95% CI: 1.64–3.63; *P* < 0.001).^[14]^

**Figure 4:**
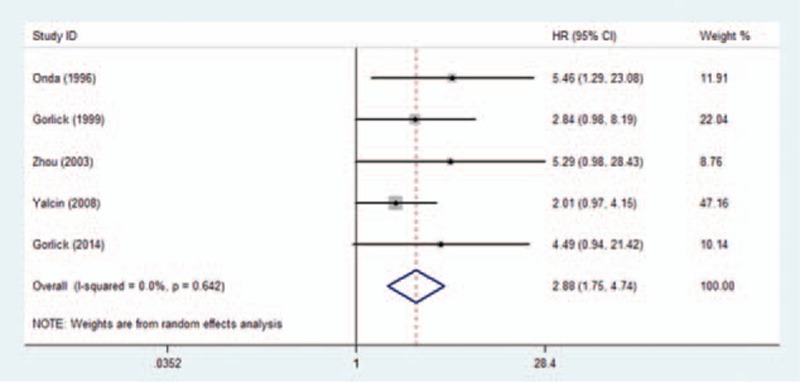
Forest plot of the association between HER-2 overexpression and overall survival in patients with localized disease at presentation.

The association between HER-2 overexpression in biopsy specimens and decreased OS was also observed in patients with localized disease at presentation (HR = 2.88; 95% CI: 1.75–4.74; *P* < 0.001) (Fig. 4). There was no statistically significant between-study heterogeneity in this subgroup (Cochran's Q *P* = 0.642; *I*^2^ = 0%).

**Figure 5:**
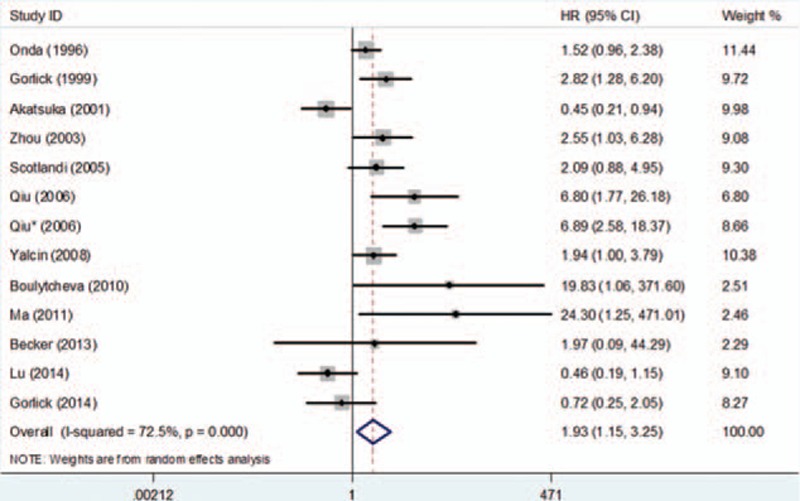
Forest plot of the association between HER-2 overexpression and disease-free survival in osteosarcoma.

A total of 13 studies reported HER-2 expression data for DFS. Compared with tumors without HER-2 expression, those with HER-2 overexpression were associated with a statistically significant decrease in DFS (HR = 1.93, 95%CI: 1.15–3.25, *P* = 0.013) (Fig. 5).

### 3.4 Sensitivity analysis and publication bias

**Figure 6:**
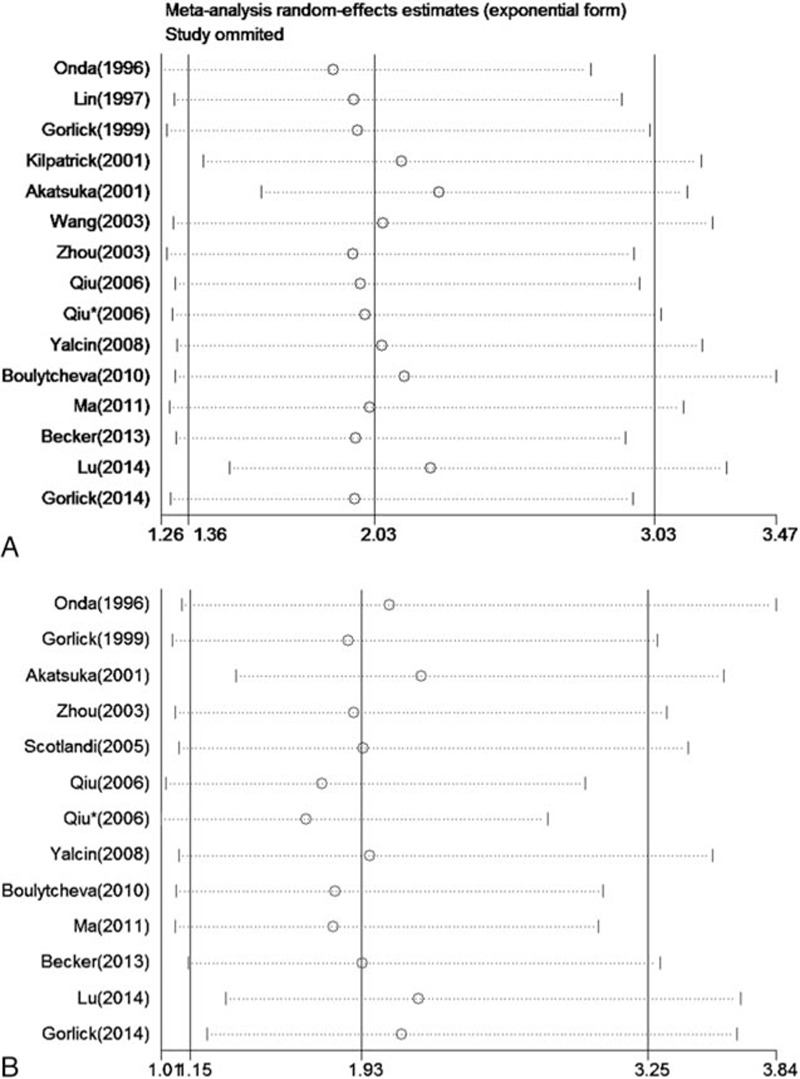
Annotation: forest plot of sensitivity analyses—(A) association between HER-2 overexpression and overall survival in osteosarcoma and (B) association between HER-2 overexpression and disease-free survival in osteosarcoma.

Sensitivity analyses suggested that the pooled HRs and relevant 95% CIs of OS and DFS maintained statistically stable when studies were omitted one by one (Fig. 6).

**Figure 7:**
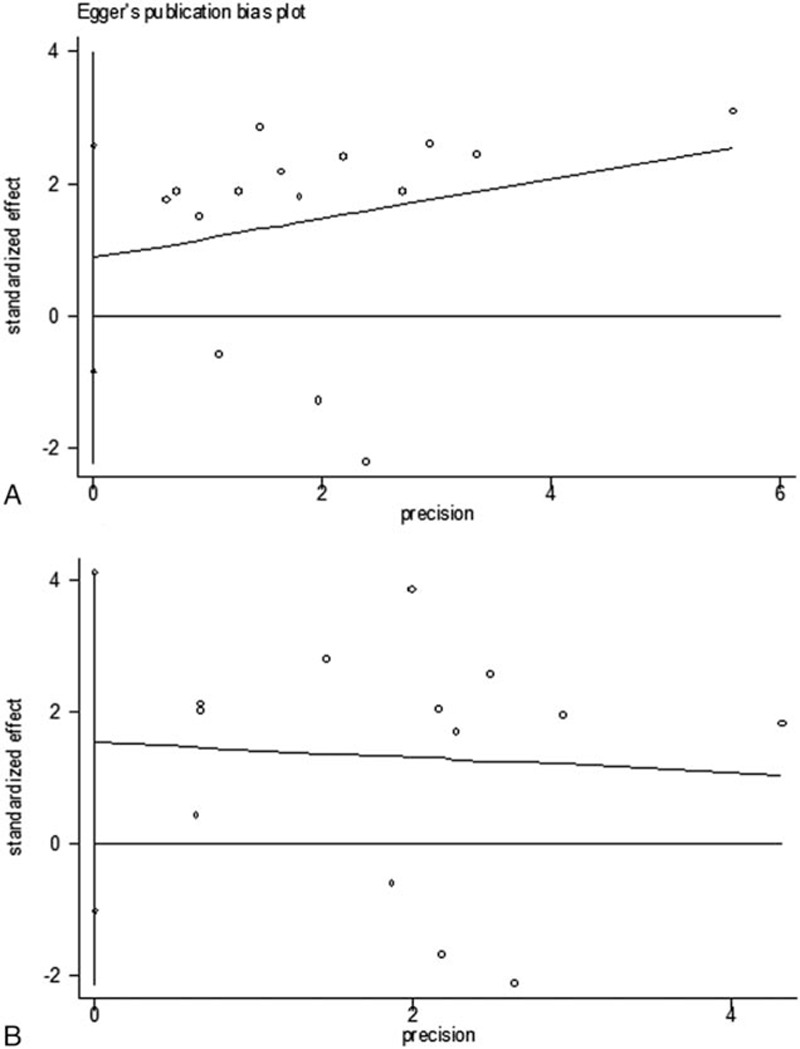
Annotation: funnel plots of publication bias—(A) publication bias for overall survival, *P* = 0.292 (Egger's) and (B) publication bias for disease-free survival, *P* = 0.218 (Egger's).

The funnel plot and Egger's test implied that no evident publication bias was observed in this meta-analysis for either OS or DFS (*P* = 0.292 and 0.218, respectively) (Fig. 7). However, funnel plots provide only hints of publication bias and not definite proof; additionally, the number of included studies was small; therefore, the results should interpreted cautiously.

## 4 Discussion

Our meta-analysis showed a significant association between HER-2 overexpression and survival in osteosarcoma, which indicated that HER-2 status should be evaluated as a prognostic marker and therapeutic target for osteosarcoma.

Therapy for osteosarcoma has hit the bottleneck. The survival of patients with metastatic osteosarcoma remains extremely poor. Presence or absence of metastatic disease and response to preoperative chemotherapy are the most critical prognostic factors used for stratification of therapy.^[29]^ It is urgent to find new and reliable markers to predict osteosarcoma prognosis.

The HER-2 oncogene is expressed widely in different tissues during the fetal period, and little is expressed in adult tissues, such as the kidney, liver, lung, skin, uterus, stomach, and colon.^[30]^ Preclinical data have demonstrated the tumorigenicity of HER-2 overexpression.^[31]^ Although it has no known ligand, HER-2 can form heterodimers with other members of the HER family, causing enhanced and prolonged intracellular signaling, including that of the mitogen-activated protein kinase (MAPK), PI3K/Akt, mTOR, Src kinase, and Signal Transducer and Activator of Transcription (STAT) pathways.^[30]^ Overexpression of HER-2 was found in the tumors of ∼15% to 20% of patients with breast cancer, and the HER-2-targeted drug trastuzumab became one of the most promising breakthroughs for treating breast cancer.^[4,5]^

Based on preclinical data, we hypothesized a worse outcome for osteosarcoma that overexpresses HER-2. A series of studies investigating the relationship between HER-2 expression and prognosis of osteosarcoma were published over the last 2 decades. In 1996, Onda et al first suggested that overexpression of HER-2 in osteosarcoma at diagnosis was associated with reduced survival.^[7]^ Unexpectedly, Akatsuka et al in 2002 proposed a correlation between overexpression of HER-2 and better outcome for osteosarcoma.^[25]^ Numerous subsequent studies reported contradictory results for both the expression status and prognostic value of HER-2. Meanwhile, the methodological variability and small sample sizes of these studies weakened their reliability. A recent meta-analysis attempted to address this issue quantitatively.^[8]^ Unfortunately, that meta-analysis neglected several studies, especially those correlating overexpression of HER-2 with a favorable prognosis for osteosarcoma and utilized a less robust technique by not taking time to an event into account when calculating outcome estimates.^[10]^ As there is no general consensus about the impact and significance of HER-2 status on survival and whether the effect is consistent among different subgroups, clinical use of HER-2 in osteosarcoma remains limited. To resolve this ongoing issue, we undertook an updated meta-analysis of all eligible studies.

The significant between-study heterogeneity for OS mostly resulted from a study conducted by Akatsuka et al that indicated a correlation between HER-2 overexpression and better prognosis. Inclusion of metastatic patients and use of less robust statistical methods in previous studies may have contributed to this difference of results.^[25]^ However, after exclusion of metastatic patients and adapting HRs and their corresponding 95% CIs as outcome estimates, the association of HER-2 overexpression and reduced survival was still stable for Onda et al's^[7]^ and Gorlick et al's^[27]^ studies, and for the pooled outcome estimate of OS of localized osteosarcoma in our meta-analysis. Furthermore, the biologic significance of Stage IIB and Stage III is not distinguishable because Stage IIB tumors may already have invisible micrometastases. No statistically significant change was found in sensitivity analyses when any of the included studies was excluded. After a careful analysis, we thought the diversity of results and between-study heterogeneity might arise from several sources. First, Akatsuka et al's study declared that they only included nonmetastatic subjects but did not state the method used to detect metastasis. Second, it had an especially high positive rate. Normally, the positive rate is inversely related to threshold, which is applicable to most of our included studies. Nevertheless, although the majority of included studies in the biopsy specimen group had a positive rate of <46%, the positive rate of Akatsuka et al's study is 63%, with the highest reported threshold (30%).^[25]^ It drew our interest that another study conducted by Kilpatrick et al^[26]^ used the same antibody and a 10% threshold, yet their positive rate was only 61.5%. The study of Lu also presented especially high positive rate. This discrepancy may result from the pathologists’ interpretation of the IHC results. Finally yet importantly, this report of Akatsuka et al was old, with the inclusion period being 1984 to 1995.

Subgroup analyses suggest a similar direction and magnitude of effect for studies investigating biopsy samples and those investigating surgical removal samples. This association between HER-2 expression and survival in osteosarcoma also was evident for nonmetastatic patients at presentation. This conclusion, to the best our knowledge, has not been highlighted previously. The *P* values of Egger's test were an index used to describe the publication bias in meta-analyses. In our investigation, the *P* values in 2 subgroup-analysis were both >0.1, which suggested that no statistically significant publication bias was found.

These findings are of clinical relevance with regard to targeted therapy for osteosarcoma. As mentioned before, the growth of osteosarcoma cell lines was inhibited when treated with trastuzumab. A phase II clinical trial using trastuzumab to treat metastatic osteosarcoma patients with HER-2-positive tumors achieved similar survival rates between the HER-2-positive and HER-2-negative groups. Given the elevated risk for patients with overexpressed HER-2, it is natural to postulate that trastuzumab was effective in extending survival for these patients.^[32]^ However, definitive evaluation of the treatment effect of trastuzumab for osteosarcoma requires randomized controlled trials of HER-2-positive patients.

Our investigation provides interesting clues, which may be useful for future studies concerning prognostic biomarkers. Among the included prognostic studies, the majority was prospective and nonblinded, and some did not utilize multivariate Cox regression analyses to predict prognosis, which could contribute to overestimation or underestimation. In the future, the methods of studies and meta-analyses regarding prognostic biomarkers should be standardized.

There were some limitations to our investigations. First, our meta-analysis was based on only 16 small cohort studies. We attempted to collect data as comprehensively as possible, but it remains possible that there are some unpublished studies, especially those that obtained negative results. Second, the antibodies and cutoff values of the included studies were not uniform, which caused the proportion of HER-2-positive samples to vary greatly across studies. These discrepancies may contribute to the heterogeneity among studies. Third, some patient characteristics, such as sex, inclusion period, and Enneking stage, were not provided in the original reports.

## 5 Conclusion

In conclusion, this meta-analysis of 16 studies demonstrated that overexpression of HER-2 in patients with osteosarcoma is significantly associated with reduced OS and DFS. HER-2 expression status should be assessed at diagnosis and after surgery as a prognostic factor. However, given the heterogeneity between studies, the results of this meta-analysis should be interpreted cautiously. Additional larger-scale and prospective studies are still needed to confirm our results.
